# Axonal swellings are related to type 2 diabetes, but not to distal diabetic sensorimotor polyneuropathy

**DOI:** 10.1007/s00125-020-05352-9

**Published:** 2021-01-23

**Authors:** Pall Karlsson, Sandra S. Gylfadottir, Alexander G. Kristensen, Juan D. Ramirez, Pedro Cruz, Nhu Le, Pallai R. Shillo, Solomon Tesfaye, Andrew S. C. Rice, Hatice Tankisi, Nanna B. Finnerup, Jens R. Nyengaard, Troels S. Jensen, David L. H. Bennett, Andreas C. Themistocleous

**Affiliations:** 1 Danish Pain Research Centre, Department of Clinical Medicine, Aarhus University, Aarhus, Denmark; 2 Core Centre for Molecular Morphology, Section for Stereology for Microscopy, Department of Clinical Medicine, Aarhus University, Aarhus, Denmark; 3 Department of Neurology, Aarhus University Hospital, Aarhus, Denmark; 4 Department of Clinical Neurophysiology, Aarhus University Hospital, Aarhus, Denmark; 5 Nuffield Department of Clinical Neurosciences, University of Oxford, Oxford, UK; 6 Faculty of Medicine, Universidade de Coimbra, Coimbra, Portugal; 7 Radboud University Medical Centre, Nijmegen, the Netherlands; 8 Diabetes Research Unit, Sheffield Teaching Hospitals NHS Foundation Trust, Sheffield, UK; 9 Pain Research Group, Department of Surgery and Cancer, Faculty of Medicine, Imperial College London, Chelsea and Westminster Hospital Campus, London, UK; 10 Pain Medicine, Chelsea and Westminster Hospital NHS Foundation Trust, London, UK

**Keywords:** Axonal swellings, Diabetes, Diabetic neuropathy, Neuropathic pain, Skin biopsy

## Abstract

**Aims/hypothesis:**

Distal diabetic sensorimotor polyneuropathy (DSP) is a common complication of diabetes with many patients showing a reduction of intraepidermal nerve fibre density (IENFD) from skin biopsy, a validated and sensitive diagnostic tool for the assessment of DSP. Axonal swelling ratio is a morphological quantification altered in DSP. It is, however, unclear if axonal swellings are related to diabetes or DSP. The aim of this study was to investigate how axonal swellings in cutaneous nerve fibres are related to type 2 diabetes mellitus, DSP and neuropathic pain in a well-defined cohort of patients diagnosed with type 2 diabetes.

**Methods:**

A total of 249 participants, from the Pain in Neuropathy Study (UK) and the International Diabetic Neuropathy Consortium (Denmark), underwent a structured neurological examination, nerve conduction studies, quantitative sensory testing and skin biopsy. The study included four groups: healthy control study participants without diabetes (*n* = 45); participants with type 2 diabetes without DSP (DSP−; *n* = 31); and participants with evidence of DSP (DSP+; *n* = 173); the last were further separated into painless DSP+ (*n* = 74) and painful DSP+ (*n* = 99). Axonal swellings were defined as enlargements on epidermal-penetrating fibres exceeding 1.5 μm in diameter. Axonal swelling ratio is calculated by dividing the number of axonal swellings by the number of intraepidermal nerve fibres.

**Results:**

Median (IQR) IENFD (fibres/mm) was: 6.7 (5.2–9.2) for healthy control participants; 6.2 (4.4–7.3) for DSP−; 1.3 (0.5–2.2) for painless DSP+; and 0.84 (0.4–1.6) for painful DSP+. Swelling ratios were calculated for all participants and those with IENFD > 1.0 fibre/mm. When only those participants with IENFD > 1.0 fibre/mm were included, the axonal swelling ratio was higher in participants with type 2 diabetes when compared with healthy control participants (*p* < 0.001); however, there was no difference between DSP− and painless DSP+ participants, or between painless DSP+ and painful DSP+ participants. The axonal swelling ratio correlated weakly with HbA_1c_ (*r* = 0.16, *p* = 0.04), but did not correlate with the Toronto Clinical Scoring System (surrogate measure of DSP severity), BMI or type 2 diabetes duration.

**Conclusions/interpretation:**

In individuals with type 2 diabetes where IENFD is >1.0 fibre/mm, axonal swelling ratio is related to type 2 diabetes but is not related to DSP or painful DSP. Axonal swellings may be an early marker of sensory nerve injury in type 2 diabetes.

**Graphical abstract:**

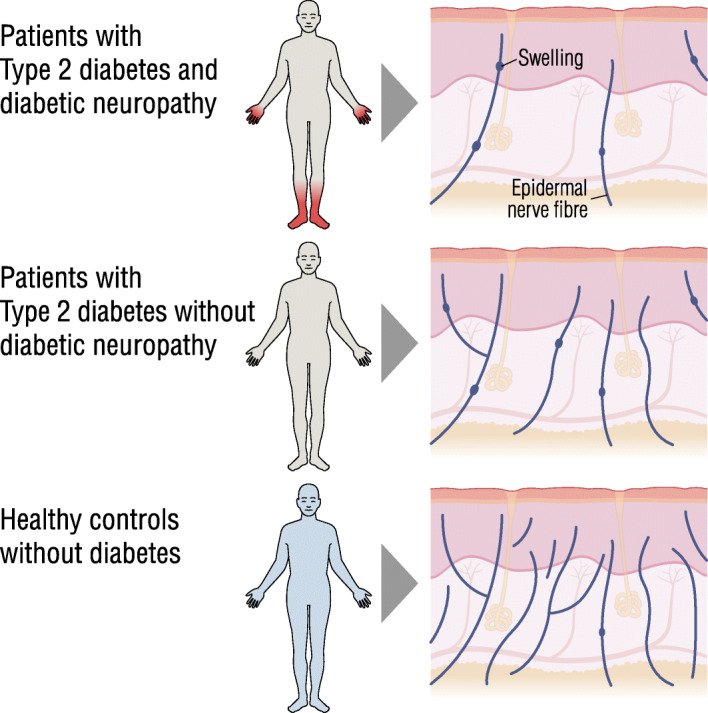

**Supplementary Information:**

The online version of this article (10.1007/s00125-020-05352-9) contains peer-reviewed but unedited supplementary material.

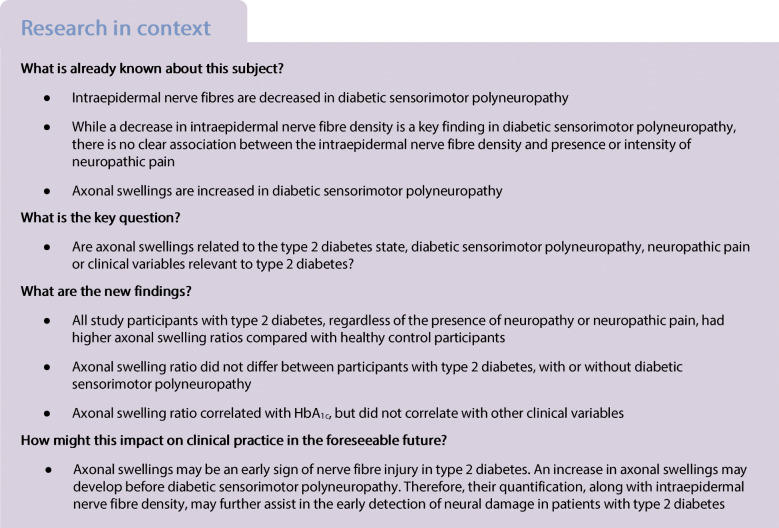



## Introduction

Diabetic sensorimotor polyneuropathy (DSP) is a common complication of diabetes, which typically presents as a distal symmetric polyneuropathy with sensory loss or pain in the feet and hands [1, 2]. As currently available treatments for neuropathic pain demonstrate modest pain relief [3], it is essential that we improve our understanding of the mechanisms that contribute to painful DSP [2, 4], and understand why some patients with DSP develop pain and others do not. Skin nerve fibre morphometric analysis offers potential insights, as differences are observed between individuals with painless and painful DSP [5].

Intraepidermal nerve fibre density (IENFD) assessment of skin biopsy samples is a validated and sensitive diagnostic tool for the assessment of small fibre neuropathies including DSP, but IENFD is considered the pathological hallmark of DSP [6–8]. There is some uncertainty as to whether IENFD differentiates between patients with painless and painful DSP, as some studies report an inverse correlation between IENFD and pain [9], while others report no correlation [10].

Morphometric analysis of nerve fibres detects a change in axonal structures termed axonal swellings, a degenerative change that contains watery axoplasm, neurofilaments and abnormal mitochondria [11]. It is postulated that axonal swellings, in particular larger swellings, precede small fibre degeneration [12, 13]. In patients with DSP the relationship of axonal swellings to polyneuropathy progression and symptoms is unclear. In one study an increase of axonal swellings was found in patients with painful DSP [5]; however, in another study axonal swellings did not differentiate between patients with painful and painless DSP [14]. In both studies, axonal swellings were higher in participants with DSP when compared with participants with diabetes but without DSP, and with healthy control (HC) participants.

Therefore, it is unclear if axonal swellings are related to type 2 diabetes, DSP or neuropathic pain.

This study investigates whether the axonal swelling ratio (axonal swellings/intraepidermal nerve fibres) is related to DSP, neuropathic pain and clinical variables in a well-characterised and comprehensively phenotyped cohort of study participants with type 2 diabetes mellitus and HC participants [10, 15].

## Methods

### Standard protocol approvals, registrations and patient consents

The data presented in this paper are derived from the Pain in Neuropathy Study (PiNS) from the UK and the International Diabetic Neuropathy Consortium (IDNC) study from Aarhus, Denmark. PiNS is a multicentre study approved by the National Research Ethics Service of the UK (No.: 10/H0706/35). The IDNC study was approved by the regional ethics committee (No.: 1-10-72-130-16). All study participants signed written consent forms, in line with the Declaration of Helsinki, before enrolment.

### Study protocol

All participants underwent a medical history review and a structured neurological examination. Study participants underwent nerve conduction studies, skin biopsy for IENFD assessment, quantitative sensory testing and diabetes-related biochemistry testing including a single HbA_1c_ test at the time of study. The clinical examination is described in detail elsewhere [10, 15]. The Toronto Clinical Scoring System (TCSS) score was calculated for all participants [16]. TCSS score is used as a surrogate measure of neuropathy severity, and was not used in the diagnosis of DSP.

### Participant selection

**Fig. 1:**
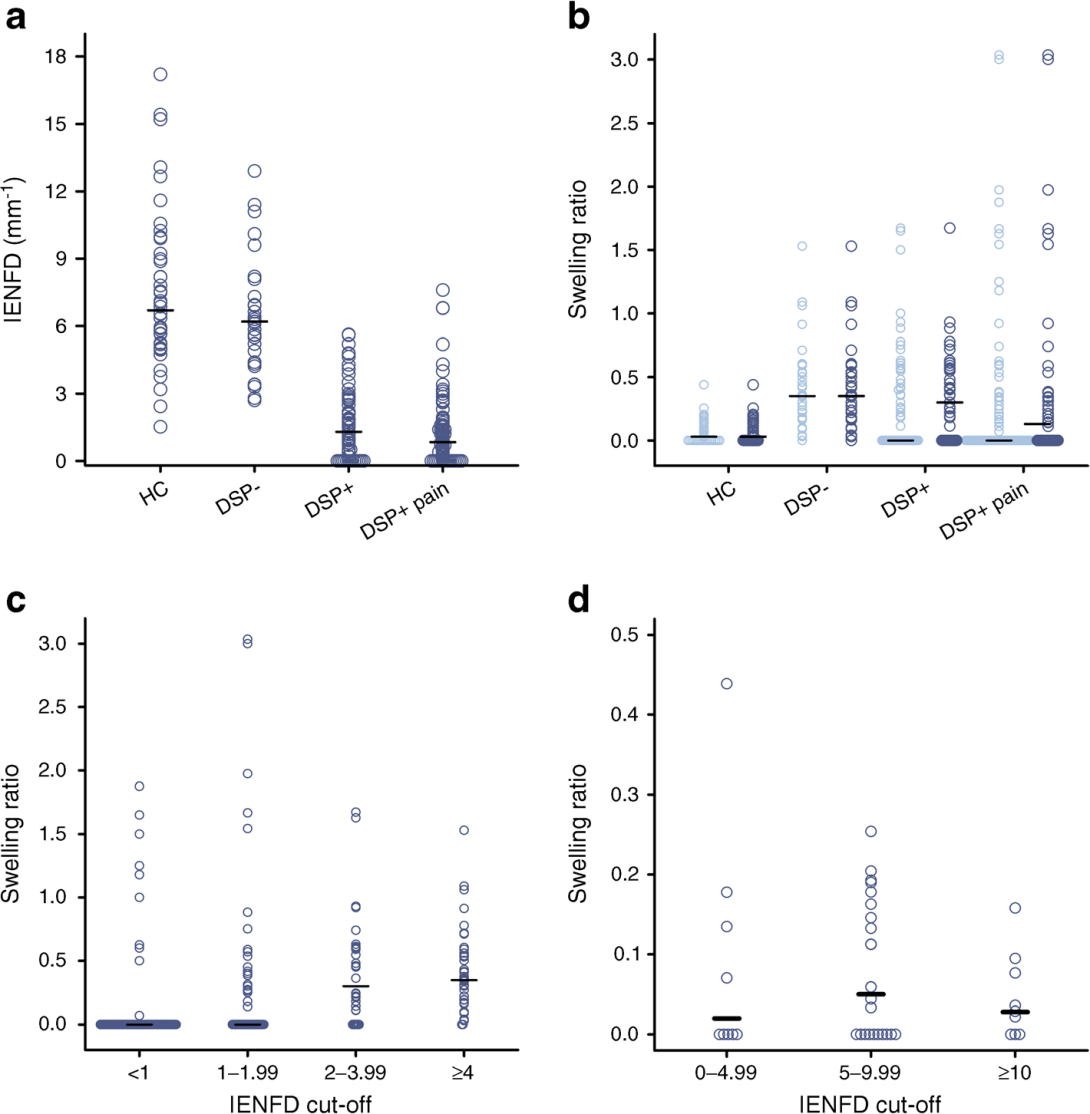
Skin biopsy results. (**a**) IENFD for all participants. (**b**) Swelling ratio for all participants (light blue) and those with IENFD >1 (dark blue). (**c**, **d**) four different IENFD cut-offs in (**c**) patients with type 2 diabetes and (**d**) HC participants

A total of 249 study participants were recruited as part of two different studies, 204 participants with type 2 diabetes and 45 HC participants without diabetes. Clinical assessment, polyneuropathy grading, neuropathic pain grading and skin staining were performed uniformly using the same methodology. A subset, 142 of the participants (57.0%), were part of the PiNS study, and all were diagnosed with type 2 diabetes [10]. The remaining 107 participants, 62 individuals with type 2 diabetes and 45 HC participants, were part of the IDNC study [15] (Electronic supplementary material [ESM] Fig. 1). A detailed description of the clinical assessment and phenotyping of the study participants can be found elsewhere and will be briefly described here [10, 15].

### Selection of IDNC participants

IDNC participants were part of a clinical study of 389 patients conducted in 2016–2018, initially recruited from a questionnaire study on neuropathy and pain of 5514 recently diagnosed individuals with type 2 diabetes from the Danish Centre for Strategic Research in Type 2 Diabetes (DD2) [15, 17]. Exclusion criteria were cognitive impairment, language difficulties and pregnancy. For more details see references [15, 17].

Of the 389 patients included at the two IDNC study sites (Aarhus and Odense), 49 were excluded with other causes of neuropathy and significant non-neuropathic pain. We then randomly selected 62 type 2 diabetes participants and 45 HC participants who were included in Aarhus where IENFD and nerve conduction study data were available (ESM Fig. 1).

We ensured that the included HC participants without diabetes, recruited from within the patients’ social circle and by invitational flyers, were as close as possible to the diabetic participants in terms of age and sex. Exclusion criteria for HC participants were diabetes, severe chronic illness, psychiatric or neurologic illness, chronic pain or intake of any pain medication within 3 days before inclusion [15]. HbA_1c_ and blood glucose were measured for all HC participants to exclude diabetes (Table 1).

**Table 1:** Demographics of study participants

Characteristic	HC (*n* = 45)	DSP− (*n* = 31)	Painless DSP+ (*n* = 74)	Painful DSP+ (*n* = 99)	*p* value
Age, years	62.2 (55.3–68.4)	62.6 (51.1–68.2)	67.9 (60.1–72.9)	66.4 (57.4–71.6)	0.008
Sex, female (%)	25 (55.6)	16 (51.6)	43 (58.1)	60 (60.6)	0.83
BMI, kg/m^2^	25.4 (22.8–28.1)	30.5 (25.3–34.3)	31.4 (26.9–35.7)	33.1 (28.1–37.9)	<0.001
Duration of type 2 diabetes, years		5.9 (3.8–7.9)	10.6 (6.0–17.5)	12.0 (6.0–18.9)	0.001
HbA_1c_					
mmol/mol	37.0 (35.0–39.0)	50.0 (46.0–57.0)	52.0 (45.7–61.5)	58.0 (50.0–69.0)	<0.001
%	5.5	6.7	6.9	7.5	
TCSS total score (0–19)	1.0 (0.0–2.0)	1.0 (0.0–3.0)	8.0 (6.0–10.0)	11.0 (8.0–14.0)	<0.001

Data are shown as median (IQR) and analysed by Kruskal–Wallis. Categorical data are shown as numbers (percentages) and analysed by Pearson χ^2^ test of association

HbA_1c_ is reported both in SI units (mmol/mol) and in percentages (NGSP: National Glycohemoglobin Standardization Program)

Detailed baseline characteristics for the original study cohorts in whole (PiNS and IDNC) are provided in previous studies [10, 15]

Missing data: BMI: 0.8%; duration of diabetes: 1.2%; HbA_1c_: 2.8%; TCSS score: 0.4%

### Selection of PiNS participants

PiNS is an observational cross-sectional multicentre study in which study participants were recruited from primary care practices in London and Oxford, and from tertiary clinics in Oxford, London and Sheffield. Patients with diabetes mellitus aged above 18 years with diagnosed DSP, or patients with symptoms and signs suggestive of DSP, were included. Exclusion criteria were pregnancy, coincident major psychiatric disorders, poor or no English language skills, severe pain at recruitment from a cause other than DSP, documented central nervous system lesions or insufficient mental capacity to provide informed consent or to complete questionnaires. The PiNS participants included in this study were participants with both IENFD and nerve conduction study data.

### Nerve conduction studies

Nerve conduction tests were performed with an ADVANCE system (Neurometrix, Waltham, MA, USA) (PiNS study) or Keypoint.Net EMG equipment (Dantec, Skovlunde, Denmark) (Aarhus) and we used conventional reusable electrodes. We performed conventional nerve conduction studies of sural nerves bilaterally and the median, peroneal and tibial nerves unilaterally [18]. If the median nerve was found to be abnormal, the ulnar nerve was examined on the same side. The results were compared with laboratory controls using *z* scores. Polyneuropathy was defined as ≥2 nerves with ≥1 abnormal measure, including at least one abnormal sural nerve [18].

### Skin biopsy

#### Skin biopsy and staining

All biopsy samples for determination of IENFD were taken in accordance with the consensus document produced by the European Federation of Neurological Societies and the Peripheral Nerve Society Guideline on the utilisation of skin biopsy samples in the diagnosis of peripheral neuropathies [7, 10]. Skin biopsies were taken 10 cm proximal to the lateral malleolus. The biopsies were fixed overnight in 2% fresh periodate-lysine-paraformaldehyde. After cryoprotection the samples were embedded in optimal cutting temperature (OCT).

For analysis under brightfield microscopy, 50 μm thin sections were used, and immunohistochemistry for protein gene product 9.5 (PGP 9.5) was performed on free-floating sections using the immunoperoxidase method. The primary antibody was a rabbit anti-PGP 9.5 antibody (1:15,000; Ultraclone, Yarmouth, Isle of Wight, UK or 1:1000; Zytomed, Dusseldorf, Germany). The secondary antibody was a biotinylated goat anti-rabbit IgG (1:400; Vector Laboratories, Burlingame, CA, USA).

#### Analysis

PGP 9.5-immunoreactive nerve fibres crossing the basal membrane of the epidermis were counted under a dry ×40 objective and a measurement of the epidermal length of the sample was obtained. IENFD was assessed using a double brightfield microscope using established counting rules and was expressed as fibres per millimetre of epidermal length. IENFD was considered abnormal if below the fifth centile for age- and sex-matched HC participants [19]. Axonal swellings were measured using newCAST stereological software Version 2019.2 (Visiopharm, Hoersholm, Denmark) and quantified using a light microscope under a ×60 oil objective (Olympus BX51 microscope, Olympus, Japan). Axonal swellings were defined as enlargements on epidermal-penetrating nerve fibres exceeding 1.5 μm in diameter and could be located anywhere on the nerve fibre, distally or proximally; i.e. located in the epidermal part of the fibre or in the dermal part [12, 20, 21]. There is currently no consensus on how to define axonal swellings, but the majority of studies define them either as enlargements exceeding 1.5 μm in diameter, as done here, or by counting swellings that exceed at least three or five times the diameter of the afferent nerve fibre. The 1.5 μm method was chosen as we believe it to be the more reliable measure of the two methods, requiring fewer measurements and calculations and thus reducing error rates. Axonal swelling ratio was obtained by dividing the number of swellings by the number of intraepidermal nerve fibres. Both IENFD and axonal swellings were counted in a blinded fashion.

### The diagnosis of definite DSP and definite painful DSP

DSP was defined according to the Toronto Diabetic Neuropathy Expert Group [22] and painful DSP according to the Neuropathic Pain Special Interest Group (NeuPSIG) criteria [23] for neuropathic pain.

The following criteria were used for neuropathy and neuropathic pain grading of participants from both cohorts.

#### No DSP

Study participants with no possible clinical neuropathy, normal nerve conduction studies and normal IENFD were defined as not having a DSP.

Possible clinical neuropathy is defined as the presence of symptoms and/or signs of neuropathy, including any one or more of the following: neuropathy symptoms (decreased sensation, positive sensory symptoms, e.g. burning, aching pain) mainly in the toes, feet or legs; decreased distal sensation; or decreased/absent ankle reflexes [22].

#### Definite DSP

Study participants with at least a possible clinical neuropathy and abnormalities on either nerve conduction studies or IENFD were defined as definite DSP.

#### Painful DSP

Definite painful DSP was defined in line with NeuPSIG criteria [23], i.e. neuropathic pain in a neuroanatomically plausible distribution, feet and/or hands, in participants with DSP.

### Statistical analysis

We used STATA version 14 (StataCorp, TX, USA) for data analysis. Data are reported as medians with IQR. Data were compared across the three groups with Kruskall–Wallis test or between two groups with Mann–Whitney *U* test. Categorical data were analysed with χ^2^ test of association. Spearman’s rank correlation analyses were performed to explore associations between swelling ratio and diabetes-related biochemical variables, e.g. HbA_1c_. Significance was set at *p* < 0.05.

All comparisons between groups were performed twice: (1) all study participants; and (2) only participants with IENFD greater than 1 fibre/mm. The IENFD cut-off of 1 was set prior to statistical analyses since it was felt from our experience that axonal swellings with lower IENFD (<1) could not be reliably counted without potential bias of our outcomes.

## Results

### Study participants

A total of 249 study participants were included. The study included four groups: (1) HC participants, *n* = 45; (2) DSP−, *n* = 31; (3) DSP+, which were divided into painful and painless DSP+, *n* = 99 and *n* = 74, respectively (ESM Fig. 1, ESM Table 1).

The characteristics of the groups are shown in Table 1. Participants in the painful DSP+ group had the highest TCSS scores, HbA_1c_ levels and BMI and the longest diabetes duration.

### IENFD and swelling ratios

**Fig. 2:**
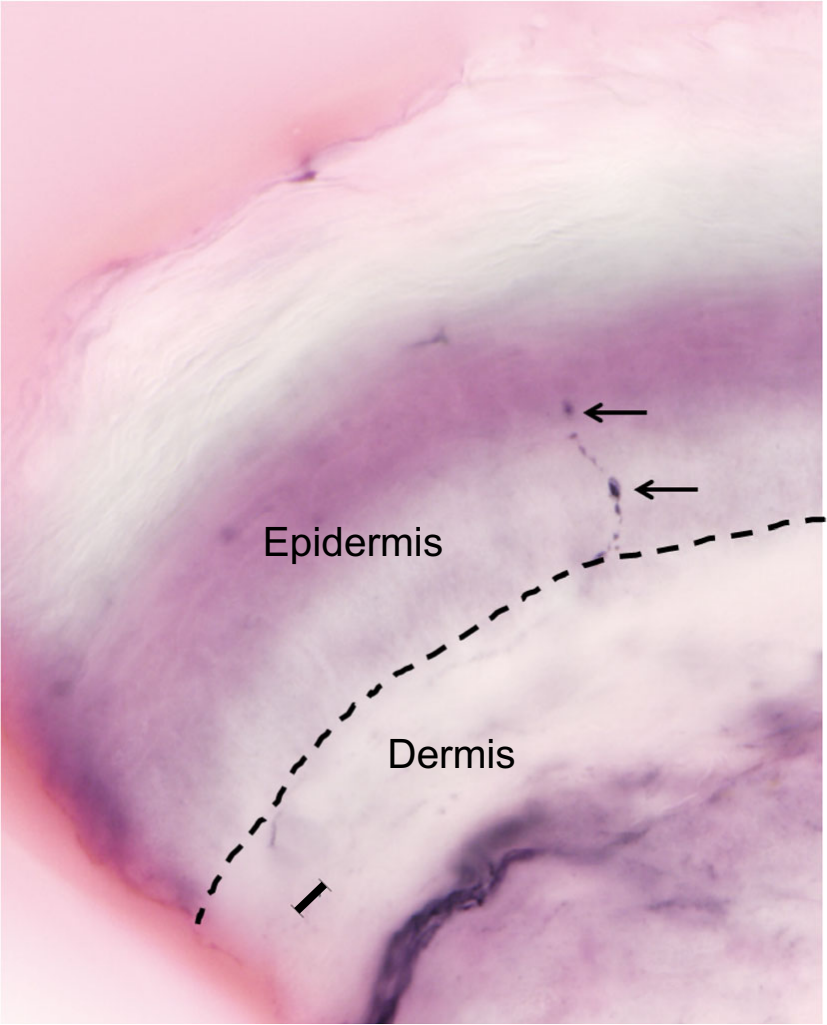
A representative image of axonal intraepidermal nerve swellings, highlighted by the arrows. The dashed line represents the division between the epidermis and dermis. Scale bar, 10 µm

There was a difference in IENFD and swelling ratios between the groups (Table 2, Fig. 1a,b, ESM Fig. 1). IENFD was decreased in DSP+ participants when compared with DSP− participants and HC participants. There was no difference between painless DSP+ and painful DSP+ participants (*p* = 0.08). Specifically, median (IQR) IENFD (fibres/mm) was: 6.7 (5.2–9.2) for healthy control participants; 6.2 (4.4–7.3) for DSP−; 1.3 (0.5–2.2) for painless DSP+; and 0.84 (0.4–1.6) for painful DSP+. The above differences remained when only participants with IENFD > 1.0 fibre/mm were included. The axonal swelling ratio was significantly higher in DSP− participants when compared with all DSP+ (painless and painful) patients and HC participants (Table 2). Figure 2 shows a representative image of axonal swellings.

**Table 2:** IENFD and swelling ratio for all study participants and for those with IENFD > 1.0 fibre/mm

Variable	HC	DSP−	Painless DSP+	Painful DSP+	*p* value
All study participants, *N* = 249					
*n*	45	31	74	99	
Swelling ratio	0.03 (0.0–0.13)	0.35 (0.17–0.59)	0.0 (0.0–0.45)	0.0 (0.0–0.24)	<0.001^b^
IENFD	6.6 (5.3–9.0)	6.2 (4.4–7.3)	1.3 (0.5–2.2)	0.8 (0.4–1.6)	<0.001^a/b^
Swellings, *n* (%)	25 (55.6)	30 (96.8)	32 (43.2)	32 (32.3)	<0.001^b^
IENFD >1 fibre/mm, *n* = 163					
*n*	45	31	43	44	
Swelling ratio	0.03 (0.0–0.13)	0.35 (0.17–0.59)	0.30 (0.0–0.57)	0.12 (0.0–0.36)	<0.001^a/b^
IENFD	6.6 (5.3–9.0)	6.2 (4.4–7.3)	2.1 (1.6–3.2)	1.7 (1.4–2.8)	<0.001^a/b^
Swellings, *n* (%)	25 (55.6)	30 (96.8)	27 (62.8)	23 (52.3)	<0.001^b^

Data are shown as median (IQR)

Swellings: *n* (%) for swellings represents the number and percentage of patients who had at least one swelling present on an intraepidermal nerve fibre

Swelling ratio: number of swellings/number of intraepidermal nerve fibres

^a^HC participants vs patients with type 2 diabetes (DSP−, painless DSP+, painful DSP+)

^b^DSP− vs painless DSP+ and painful DSP+

If the IENFD is very low (as can be the case in DSP+), it is not possible to reliably calculate the swelling ratio because so few intraepidermal nerve fibres are sampled. A total of 86 study participants (out of 249) had IENFD ≤ 1 fibre/mm: 31 (41.9%) painless DSP+ participants and 55 (55.6%) painful DSP+ participants. When the participants with IENFD ≤ 1 fibre/mm were excluded, the median swelling ratios for painless DSP+ and painful DSP+ participants increased from 0.0 to 0.3 and 0.13, respectively (Table 2, Fig. 1b). Therefore, the axonal swelling ratio was higher in study participants with type 2 diabetes, irrespective of whether DSP was present, when compared with HC participants. The axonal swelling ratio increased in participants with type 2 diabetes with higher IENFD (Fig. 1c), but not in the HC participants (Fig. 1d).

Table 3 shows the correlation between clinical variables and swelling ratio in the study participants. The difference observed when including all study participants disappears when excluding patients with IENFD ≤ 1, leaving only a weak correlation to HbA_1c_; this suggests that the difference was driven by the fact that participants with low IENFD are unlikely to have swellings present (Table 3). Specifically, the axonal swelling ratio correlated weakly with HbA_1c_ (*r* = 0.16, *p* = 0.04), but did not correlate with the Toronto Clinical Scoring System (surrogate measure of DSP severity), BMI or type 2 diabetes duration.

**Table 3:** Correlation between swelling ratio and clinical characteristics, for all 249 study participants and the 163 with IENFD >1.0 fibre/mm

Characteristic	All study participants *N* = 249 Spearman’s ρ/*p*	IENFD >1.0 fibre/mm *n* = 163 Spearman’s ρ/*p*
Sex, female	0.02/0.70	0.02/0.83
Age, years	−0.03/0.68	0.04/0.60
BMI, kg/m^2^	−0.07/0.22	0.06/0.45
HbA_1c_, mmol/mol	−0.09/0.17	0.16/0.04
TCSS score	−0.15/0.02	0.07/0.40
Duration of type 2 diabetes, years	−0.18/0.01	−0.17/0.07
IENFD	0.36/<0.001	0.04/0.60

## Discussion

In this study, we addressed the question of whether axonal swellings are related to type 2 diabetes, DSP or neuropathic pain. Our key findings are that where IENFD > 1.0 fibre/mm, the axonal swelling ratio is increased in type 2 diabetes when compared with HC participants; axonal swelling ratio did not differ between study participants with or without painless DSP+, or between painless DSP+ and painful DSP+. There was a weak correlation between axonal swelling ratios and HbA_1c_ but not other clinical variables. These findings indicate that patients with type 2 diabetes with preserved intraepidermal nerve fibres have more axonal swellings compared with HC participants; however, the presence of axonal swellings is not associated with DSP or neuropathic pain. This suggests that axonal swellings are pathological and an early marker of sensory neuron injury in type 2 diabetes.

Axonal swellings have been defined and quantified in different ways. Hence, direct comparison between studies is difficult. Axonal swellings in this study were defined by absolute measurements of 1.5 μm [14], and not by their size relative to adjoining nerve fibres [5, 13]. We found, as did Cheung et al. [14], no axonal swelling ratio difference between those with painless DSP and those with painful DSP. In contrast, the studies that defined axonal swellings relative to adjoining nerve fibres, as 3–5 times the diameter of the afferent nerve fibre, found an association between axonal swellings and symptomatology [5, 13]. Therefore, studies that used axonal swellings relative to axon fibre saw an association with symptomatology, while studies using an absolute size cut-off did not. The reason for these differences is unclear. Such a finding highlights the importance of reaching a consensus on swelling definition and how this should be quantified and measured. We used an absolute cut-off value of 1.5 μm as it is more reliable than multiple measurements of the afferent fibre. Indeed, electron microscopy studies show that C fibre diameters in 95% of HC participants are less than 0.5 μm, with some as low as 0.2 μm. Therefore, in our study the axonal swellings are at least three times the upper limits of normal of healthy C fibres [24]. Lastly, the discrepancy between our findings and other studies may be due to differences in clinical variables such as age or diabetes duration [5, 13].

Our findings may indicate that axonal swellings are related to type 2 diabetes rather than to DSP and neuropathic symptoms. There is a weak correlation to single-point HbA_1c_ and no correlation to type 2 diabetes duration or BMI. It is likely that axonal swellings are a sign of nerve injury. A longitudinal study tracking the development of DSP, symptoms and axonal swellings is needed to determine the natural history of axonal swellings and their relationship to DSP and neuropathic pain. It is also not clear what pathological process causes axonal swellings.

Axonal swellings are present in skin biopsies from HC participants, but are clearly higher in patients with polyneuropathies [5, 14, 20]. Axonal swellings are described in patients with AIDS/HIV [11, 12], pure small fibre neuropathy [21], distal symmetric polyneuropathy of various aetiologies [20], idiopathic neuropathic pain [11–14, 20], bortezomib-induced neuropathy [25] and amyotrophic lateral sclerosis (ALS) [26, 27], and in myelinated nerve fibres of patients with Charcot–Marie–Tooth disease [28]. Axonal swellings may be associated with dysfunctional axonal transport and future nerve fibre loss, or may even be a potential biomarker of axonal regeneration [5, 12]. Detailed electron microscopy analysis of axonal swellings could yield insights into their potential mechanism, but needs technical modifications in how skin biopsies are processed.

This study has a number of strengths and limitations. Our study consisted of a large cohort of individuals with type 2 diabetes that were well phenotyped. All participants were examined by a clinician and underwent multiple tests to confirm the presence or absence of polyneuropathy and neuropathic pain. Our approach of clinical assessment followed by confirmatory investigations is the current gold standard and most rigorous approach for the diagnosis of polyneuropathy and neuropathic pain. Limitations include the retrospective study design, participants from two distinct study sites and unequal distribution among subgroups. The axonal swelling ratio was only different when IENFD >1 fibre/mm, thus limiting its role in the diagnosis of DSP, where IENFD is often lower. However, this does not negate the importance of studying axonal swellings to improve our understanding of early morphological abnormalities in diabetic neural dysfunction. Lastly, the findings in this study are applicable to type 2 diabetes only, as no patients with type 1 diabetes were included.

## Conclusion

We have shown in a large cohort of well-characterised participants that skin biopsies from participants with type 2 diabetes have higher axonal swelling ratio compared with skin biopsies from healthy study participants, independent of DSP, if IENFD >1 fibre/mm. Axonal swelling ratio was weakly associated with HbA_1c_ levels but not with neuropathy severity, BMI or diabetes duration.

## Supplementary Information

ESM 1 (PDF 167 kb)

## Data Availability

All data are available from the corresponding author upon reasonable request.

## Abbreviations

DSP: Diabetic sensorimotor polyneuropathy
DSP+: Participants with DSP
DSP−: Participants without DSP
IDNC: International Diabetic Neuropathy Consortium
IENFD: Intraepidermal nerve fibre density
NeuPSIG: Neuropathic Pain Special Interest Group
PGP 9.5: Protein gene product 9.5
PiNS: Pain in Neuropathy Study
TCSS: Toronto Clinical Scoring System
