# Supplementary material for: Axonal swellings are related to type 2 diabetes, but not to distal diabetic sensorimotor polyneuropathy
Source: Diabetologia. 2021 Jan 23;64(4):923–31. doi: 10.1007/s00125-020-05352-9 (PMC7940290; doi:10.1007/s00125-020-05352-9)
Supplement: Supplementary file 1 — (PDF 167 kb) [file 125_2020_5352_MOESM1_ESM.pdf]

Electronic supplementary material

Table ESM 1. The study group of 249 patients. Diagnosis and centers.

	**PiNS**	**IDNC**	**Total**
HC	0	45	**45**
DSP-	11	20	**31**
Painless DPN+	51	23	**74**
Painful DPN+	80	19	**99**
**Total**	**142**	**107**	**249**

Patients fulfilling the diagnostic criteria of: Definite DPN, definite painful DPN or no DPN and valid data on IENFD and swellings.

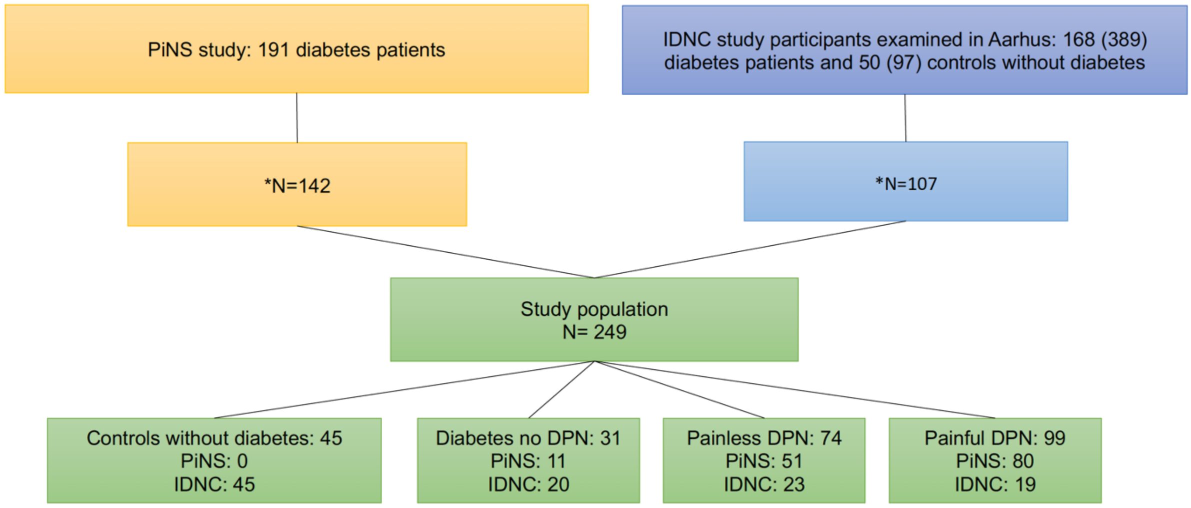

Figure ESM 1. Inclusion diagram of the two studies that constitute the total cohort of this study.

*Inclusion criteria for the current study: a diagnosis of definite DPN or painful DPN, data on both IENFD and swellings. The IDNC participants included in this study were randomly selected among participants recruited at Aarhus University, Denmark, who fulfilled the inclusion criteria.
